# Neutrophil levels correlate with quantitative extent and progression of fibrosis in IPF: results of a single-centre cohort study

**DOI:** 10.1136/bmjresp-2023-001801

**Published:** 2023-10-10

**Authors:** Andrew Achaiah, Emily Fraser, Peter Saunders, Rachel K Hoyles, Rachel Benamore, Ling-Pei Ho

**Affiliations:** 1 Translational Immunology Discovery Unit, Weatherall Institute of Molecular Medicine, University of Oxford, Oxford, UK; 2 Oxford Interstitial Lung Disease Service, Oxford University Hospitals NHS Foundation Trust, Oxford, UK; 3 Thoracic Radiology Department, Oxford University Hospitals NHS Foundation Trust, Oxford, UK

**Keywords:** Innate Immunity, Interstitial Fibrosis

## Abstract

**Background:**

Idiopathic pulmonary fibrosis (IPF) is a progressive fibrotic lung disease with poor prognosis. Clinical studies have demonstrated association between different blood leucocytes and mortality and forced vital capacity (FVC) decline. Here, we question which blood leucocyte levels are specifically associated with progression of fibrosis, measured by accumulation of fibrosis on CT scan using a standardised automated method.

**Methods:**

Using the Computer-Aided Lung Informatics for Pathology Evaluation and Rating CT algorithm, we determined the correlation between different blood leucocytes (<4 months from CT) and total lung fibrosis (TLF) scores, pulmonary vessel volume (PVV), FVC% and transfer factor of lung for carbon monoxide% at baseline (n=171) and with progression of fibrosis (n=71), the latter using multivariate Cox regression.

**Results:**

Neutrophils (but not monocyte or lymphocytes) correlated with extent of lung fibrosis (TLF/litre) (r=0.208, p=0.007), PVV (r=0.259, p=0.001), FVC% (r=−0.127, p=0.029) at baseline. For the 71 cases with repeat CT; median interval between CTs was 25.9 (16.8–39.9) months. Neutrophil but not monocyte levels are associated with increase in TLF/litre (HR 2.66, 95% CI 1.35 to 5.25, p=0.005).

**Conclusion:**

Our study shows that neutrophil rather than monocyte levels correlated with quantifiable increase in fibrosis on imaging of the lungs in IPF, suggesting its relative greater contribution to progression of fibrosis in IPF.

**WHAT IS ALREADY KNOWN ON THIS TOPIC**

- Idiopathic pulmonary fibrosis (IPF) is a progressive fibrotic condition. Recently, several human studies have implicated blood leucocyte levels (monocyte, neutrophil and lymphocyte) with forced vital capacity decline and mortality. However, direct association between leucocytes and progression of fibrosis using quantitative CT analysis has not been explored.

**WHAT THIS STUDY ADDS**

- This study explored the association between blood monocytes, neutrophils and lymphocytes against increase in fibrosis over time, measured using a quantitative CT algorithm, Computer-Aided Lung Informatics for Pathology Evaluation and Rating. We show that levels of blood neutrophil and lymphocytes but not monocytes were associated with greater risk of progression of fibrosis.

**HOW THIS STUDY MIGHT AFFECT RESEARCH, PRACTICE OR POLICY**

- Our study shows that neutrophil rather than monocyte levels correlated with quantifiable increase in fibrosis on imaging of the lungs in IPF, suggesting its relative greater contribution to progression of fibrosis in IPF.

## Introduction

Idiopathic pulmonary fibrosis (IPF) is a progressive fibrotic condition. Animal studies have implicated innate immune cells, particularly monocytes and macrophages in the pathogenesis of IPF.^1^ This is supported by several clinical studies which showed that higher blood monocytes levels are associated with mortality in IPF.^2^ Blood monocyte levels also correlated with the extent of fibrosis on CT scan,^3^ and Kreuter *et al* also found an association with a composite measure of IPF outcome (forced vital capacity (FVC) decline, 6 min walk distance reduction, acute exacerbation and/or mortality).^4^ Moreover, we (and others) have shown that the neutrophil:lymphocyte ratio (NLR) is also a predictor of mortality and FVC decline,^5 6^ and patients with higher levels of neutrophils were more likely to progress from indeterminate for Usual Interstitial Pneumonia (UIP) CT pattern to UIP pattern and a clinical diagnosis of IPF.^7^

However, mortality and FVC decline may not accurately reflect progression of fibrosis. Death can be due to other causes, for example, cardiovascular diseases, especially in the elderly population of IPF. Although FVC decline of >10% in IPF is the validated measure of disease progression predictive of mortality,^8^ and serial FVC change the recommended monitoring variable in international guidelines,^9^ this global metric may not accurately reflect regional morphological changes indicative of progression of fibrosis. In fact a recent large study suggests that FVC decline in IPF patients is heterogeneous and may have little correlation with increase in fibrosis in some patients.^10^

The Computer-Aided Lung Informatics for Pathology Evaluation and Rating algorithm (CALIPER) is an automated quantitative CT application which characterises lung parenchymal features on volumetric high-resolution CT (HRCT), thus quantifying abnormal lung.^11^ In IPF studies it is highly predictive of mortality,^12^ but also enhances risk stratification and cohort enrichment for trial end points.^13 14^ Until now, it has not yet been used to explore leucocyte association with progression of fibrosis.

The aim of the study is twofold—(1) to assess if leucocyte levels are linked to progression in fibrosis per se and (2) to explore which leucocytes demonstrate greatest association with fibrosis. We used CALIPER to provide a standardised automated, method of quantifying lung fibrosis.^11^

## Methods

### Study design and patients characteristics

We performed a retrospective analysis of a cohort of patients with IPF, who presented to the Oxford Interstitial Lung Disease (ILD) Service between September 2016 and November 2021. All patients with a multidisciplinary team (MDT) diagnosis of IPF,^15^ and a CALIPER-compatible HRCT (non-contrast, supine, volumetric HRCT scan) were included.

We asked two main questions—(1) what is the correlation between contemporaneous blood leucocyte levels (within 4 months of the first CT) and baseline amount of lung fibrosis and (2) which blood leucocyte levels (neutrophils, lymphocytes, monocytes and their derived ratios) correlated with progression in amount of lung fibrosis. Association between blood leucocytes and FVC decline and mortality were also examined as comparator end points.

### Blood leucocyte measurement

Neutrophil, lymphocyte and monocyte levels captured from standard clinical ‘full blood count’ analysis within 4 months of initial CT (CT1). NLR, monocyte:lymphocyte ratio (MLR) and Systemic Inflammation Response Index (SIRI) (neutrophil×monocyte/lymphocyte) were calculated as described previously.^5^

### CT scans

Non-contrast, supine, volumetric HRCT scan were acquired using a 64-detector row CT scanner. Images were reconstructed using a high spatial resolution algorithm. Non-contrast, volumetric, HRCT scans for appropriate subjects were acquired (0.625 mm slice thickness at an interval of 0.625 mm).

### Computer-Aided Lung Informatics for Pathology Evaluation and Rating

Full CALIPER data acquisition and processing are described in online supplemental methods. Briefly, classification of parenchymal features was applied to 15×15×15 voxel volumes of interest (VOI) using texture analysis and computer-based algorithmic interpretation of volumetric histogram signature mapping features.^11^ CALIPER evaluation included characterisation and quantification of each VOI into one of five radiological parenchymal categories: normal lung, hyperlucent, ground glass opacity (GGO), reticular opacity and honeycombing. Parenchymal features were expressed as a relative percentage of CALIPER-derived total lung volume. Total lung fibrosis (TLF) represented the sum of GGO, reticular and honeycomb percentages.^14^ We included GGO as these had been reported by our radiologists to be found in areas of reticulation or traction bronchiectasis at MDTs and designated as fine fibrosis in keeping with agreed radiological assessments.^16^

10.1136/bmjresp-2023-001801.supp1 Supplementary data

Assessment of pulmonary vessel volume (PVV) was also performed prior to pulmonary vessel extraction from lung parenchyma using a multiscale tubular structure enhancement filter.^17^ This assessment excluded large vessels at the hilum of the lung. The PVV was calculated as absolute volume (cm^3^) and expressed as percentage of total CALIPER-derived lung volume.

The key variables were TLF (the sum of reticulation, GGO and honeycomb, as % of CALIPER-derived lung volume), TLF/litre (TLF expressed as % of lung volume) and PVV. TLF/litre was used to account for difference in lung volume on serial scans with progression in fibrosis.^18^ %change in fibrosis is (CT2 TLF/litre–CT1 TLF/litre)/CT1 TLF/litre×100.

### Lung function tests

Baseline spirometry (forced expiratory volume in 1 s and FVC), transfer factor of lung for carbon monoxide (TLco) and Composite Physiological Index (CPI)^19^ within 3 months of CT were recorded; all as % predicted for age, height and sex. Annualised change in lung function was calculated as the relative (%) change in absolute value divided by time (years) between tests.

### Statistical analysis

Pearson correlation was used to explore association between blood leucocytes with baseline CALIPER variables and pulmonary function tests. Cox proportional hazard modelling was employed to test association between each blood leucocyte level (monocytes, lymphocytes and neutrophils) and outcomes of disease progression—(1)≥7.8% increase in TLF/litre (7.8% being the lower limit of upper tertile) (model A), (2) ≥10% increase in TLF/litre (10% as an arbitrary value) (model B) and (3) ≥10% FVC decline between CT1 and CT2 (model C). Median and upper quartiles for model A were also tested in preliminary modelling but no significant correlation was found. Harrell’s Concordance index (C-index) was used to assess model strength, describing how well a model can discriminate between two survival distributions.^20^ Kaplan-Meier analysis (log rank test) was used to evaluate time to all-cause mortality from first CT. A censoring time of 1 January 2022 was applied.

All analyses were performed using GraphPad Prism V.9.2 (La Jolla, California, USA) or SPSS V.27 (IBM).

## Results

### Patients

A total of 171 eligible patients were identified. Of these, n=71 had at least one further follow-on HRCT (the CT with the longest interval from the first was selected). Demographics are shown in table 1. Median interval between CT1 and CT2 was 25.9 (16.8–39.9) months. Median time from first CT to death was 26.2 months (12.4–43.4) vs 45.7 (34.6–61.5) in those surviving to censoring date.

**Table 1:** Baseline characteristics for patients, at the point of first CT used for CALIPER analysis.

	All patients (n=171)	Patients for analysis of progression (n=71)
Demographics		
Age at CT in years	75.1 (70.1–81.1)	73.6 (68.3–79.3)
Female, n (% of cohort)	17 (9.9)	6 (8)
Male, n (% of cohort)	154 (90.1)	65 (92)
Smoking status		
Never smoker, n (% of cohort)	47 (27.5)	21 (29.6)
Ex-smoker, n (% of cohort)	89 (52.3)	29 (40.8)
No data, n (% of cohort)	35 (20.2)	21 (29.6)
CALIPER parenchymal features		
Total lung fibrosis or TLF, as % of lung volume	14% (8–22)	13% (7–19)
Low attenuation areas, as % of lung volume	1% (0–2)	1% (0–3)
Total ground glass, as % of lung volume	5% (3–13)	5% (2–10)
Total reticular, as % of lung volume	5% (3–9)	5% (3–8)
Total honeycombing, as % of lung volume	1% (0–1)	0% (0–1)
CALIPER pulmonary vessel volume		
Total PVV in cm^3^	160 (128–201)	168 (134–202)
Total PVV as % of lung volume	4.02% (3.02–5.21)	4.60% (3.04–6.62)
Lung function		
FEV1, litre	2.19 (1.83–2.68)	2.34 (1.93–2.83)
%FEV1	81.2% (70.9–92.2)	84.7% (71.53–91.15)
FVC, litre	2.8 (2.28–3.365)	2.91 (2.35–3.56)
%FVC	76.57% (66.6–91.15)	76.44% (68.9–92.37)
FEV1: FVC	80.6 (75.0–86.1)	82.1 (75.1–88.6)
TLCO, SI	4.56 (3.49–5.48)	4.9 (3.87–5.80)
%TLCO	56.85% (49.5–67)	60.05% (51.1–71)
CPI Score	67.48 (61.05–73.12)	65.36 (59.64–71.25)
Blood leucocytes		
Monocyte ×10^3^/μL	0.67 (0.58–0.84)	0.66 (0.55–0.8)
Neutrophil ×10^3^/μL	4.90 (3.85–6.53)	4.87 (3.85–6.45)
Lymphocyte ×10^3^/μL	1.62 (1.31–2.23)	1.64 (1.43–2.27)
MLR	0.37 (0.32–0.55)	0.37 (0.29–0.5)
NLR	2.70 (2.08–4.08)	2.69 (2.06–3.61)
SIRI	1.79 (1.4–3.08)	1.76 (1.27–2.63)
Comorbidity		
Reflux, n (% of cohort)	54 (39.1)	25 (39.7)
PHT, n (% of cohort)	11 (8.0)	4 (6.3)
IHD, n (% of cohort)	28 (20.3)	15 (23.8)
Cardiomyopathy, n (% of cohort)	6 (5.8)	1 (2)
Arrhythmia, n (% of cohort)	9 (6.5)	2 (3.2)
Hypertension, n (% of cohort)	29 (21)	16 (25.4)
PE, n (% of cohort)	3 (2.2)	2 (3.2)
COPD/emphysema, n (% of cohort)	8 (7.7)	4 (8.2)
Type II diabetes mellitus, n (% of cohort)	15 (10.9)	7 (11.1)
Antifibrotics		
Antifibrotic use, n (% of cohort)	69 (40.3)	36 (57.1)

All values are median (IQR) unless stated. CPI as calculated by Wells *et al*.^19^ Forty-nine per cent of the cohort had ‘probable UIP’ as defined by the 2018 American Thoracic Society (ATS) criteria (all had clinical IPF from MDT diagnosis), explaining why some patients do not have honeycomb. Low attenuation areas represent emphysematous areas.

CALIPER, Computer-Aided Lung Informatics for Pathology Evaluation and Rating; CO, carbon monoxide; COPD, Chronic obstructive pulmonary disease; CPI, Composite Physiological Index; FEV1, forced expiratory volume in 1s; FVC, forced vital capacity; IHD, Ischaemic heart disease; IPF, idiopathic pulmonary fibrosis; MDT, multidisciplinary team; MLR, monocyte:lymphocyte ratio; NLR, neutrophil:lymphocyte ratio; PHT, pulmonary hypertension; PVV, pulmonary vessel volume; SI, standard international; SIRI, Systemic Inflammation Response Index; TLCO, transfer factor of lung for carbon monoxide.

### Association between blood leucocyte levels and baseline CALIPER and lung function measures

At baseline (CT1, n=171), there were significant correlations between baseline % predicted FVC, TLCO and CPI, and TLF, TLF/litre and PVV (table 2). In terms of correlation between leucocytes and CALIPER variables and lung function, there was significant correlation between neutrophil levels and TLF/litre (r=0.208, p=0.007), total PVV (r=0.259, p=0.001) and FVC (r=−0.127, p=0.029) (table 3). No other leucocyte levels or their derived measures showed significant corelations with CALIPER or lung function at baseline (table 3 and online supplemental table S1).

**Table 2:** Association between lung function and CALIPER parameters (Pearson’s correlation) at baseline; n=171 patients.

	FVC%		TLCO%		CPI	
CALIPER baseline metrics	r	P value	r	P value	r	P value
Lung volume (litre)	0.634	**<0.0001**	0.411	**<0.0001**	0.112	0.156
TLF (%)	0.392	**<0.0001**	0.461	**<0.0001**	0.275	**<0.0001**
TLF (/litre)	0.430	**<0.0001**	0.444	**<0.0001**	0.246	**0.002**
Low attenuation areas (% of total lung volume)	0.217	0.061	0.107	0.177	0.066	0.404
GGO (% of total lung volume)	0.286	**<0.0001**	0.316	**<0.0001**	0.175	**0.005**
Reticulation (% of total lung volume)	0.306	**<0.0001**	0.407	**<0.0001**	0.285	**<0.0001**
Honeycomb (% of total lung volume)	0.039	0.534	0.087	0.173	0.134	**0.033**
PVV (cm^3^)	0.068	0.284	0.322	**<0.0001**	0.253	**<0.0001**
PVV (%)	0.448	**<0.0001**	0.473	**<0.0001**	0.262	**<0.0001**

Values in bold signifies p<0.05.

CALIPER, Computer-Aided Lung Informatics for Pathology Evaluation and Rating; CPI, Composite Physiological Index; FVC, forced vital capacity; GGO, ground glass opacity; PVV, pulmonary vessel volume; TLCO, transfer factor of lung for carbon monoxide; TLF, total lung fibrosis.

**Table 3:** Correlation of blood leucocytes with CALIPER parameters and lung function (n=171 patients, Pearson’s correlation).

Blood leucocytes	Monocytes		Neutrophils		Lymphocytes	
Baseline metrics	r	P value	r	P value	r	P value
CALIPER scores						
TLF (/litre)	0.040	0.606	0.208	**0.007**	0.136	0.082
PVV (%)	0.110	0.164	0.259	**0.001**	0.019	0.809
Lung function tests						
FVC%	0.096	0.101	0.127	**0.029**	0.014	0.814
TLCO%	0.104	0.080	0.104	0.079	0.085	0.155
CPI	0.103	0.081	0.091	0.127	0.022	0.708

Values in bold signifies p<0.05.

CALIPER, Computer-Aided Lung Informatics for Pathology Evaluation and Rating; CPI, Composite Physiological Index; FVC, forced vital capacity; PVV, pulmonary vessel volume; TLCO, transfer factor of lung for carbon monoxide; TLF, total lung fibrosis.

### Association between blood leucocyte levels and progression of fibrosis

Neutrophil count was significantly higher in cases demonstrating progression of fibrosis as defined by ∆TLF>10%/litre (4.87 (3.86–5.66) vs 4.84 (3.85–6.45), p=0.043). No other differences in leucocyte measure between cases with disease progression and stability was identified (online supplemental table 2).

In model A of Cox proportional hazard analysis, neutrophil levels (HR 1.81, 95% CI 1.10 to 2.99, p=0.020) were significantly associated with increase in TLF/litre≥7.8% (table 4). Lower lymphocyte levels were also significantly associated with TLF/litre≥7.8% (HR 0.26, 95% CI 0.08 to 0.91, p=0.034). Similar findings were observed in model B (where outcome was TLF/litre ≥10%), except that lower lymphocyte levels were not significantly associated with progression of >10%/Litre. In both models A and B, there was a trend of higher monocyte levels with progression of fibrosis, but this was not statistically significant.

**Table 4:** Cox proportional hazard analysis for progression of lung fibrosis.

Multivariate cox regression	HR	95% CI	P value
Model A: Increase in TLF≥7.8%/litre between CT1 and CT2			
Age at CT	0.96	0.88 to 1.05	0.404
Male	0.03	0.01 to 0.56	**0.019**
Change in lung volume (%)	0.86	0.80 to 0.93	**<0.001**
Total lung fibrosis (/litre) on first CT	1.12	1.05 to 1.20	**0.001**
Total PVV (%)	1.03	1.01 to 1.05	**0.014**
Low attenuation areas (%)	1.06	0.94 to 1.20	0.318
Monocyte (×10^3^/μL)	1.35	0.10 to 18.38	0.820
Neutrophil (×10^3^/μL)	1.81	1.10 to 2.99	**0.020**
Lymphocyte (×10^3^/μL)	0.26	0.08 to 0.90	**0.034**
Harrell’s Index of concordance=0.91			
Model B: Increase in TLF≥10% /litre between CT1 and CT2			
Age at CT	0.93	0.84 to 1.04	0.208
Male	0.02	0.02 to 0.46	**0.016**
Change in lung volume (%)	0.82	0.73 to 0.92	**<0.001**
Total lung fibrosis (/litre) on first CT	1.13	1.03 to 1.25	**0.013**
Total PVV (%)	1.04	1.01 to 1.07	**0.037**
Low attenuation areas (%) (%)	1.10	0.97 to 1.25	0.142
Monocyte (×10^3^/μL)	2.37	0.09 to 62.20	0.604
Neutrophil (×10^3^/μL)	2.66	1.35 to 5.25	**0.005**
Lymphocyte (×10^3^/μL)	0.30	0.07 to 1.24	0.096
Harrell’s Index of concordance=0.93			
Model C: Decline in FVC>10% between CT1 and CT2			
Age at CT	1.01	0.92 to 1.11	0.856
Male	0.40	0.07 to 2.31	0.308
%FVC baseline	0.96	0.92 to 0.99	**0.049**
Monocyte (×10^3^/μL)	0.96	0.03 to 28.51	0.979
Neutrophil (×10^3^/μL)	1.17	0.91 to 1.52	0.222
Lymphocyte (×10^3^/μL)	0.71	0.30 to 1.71	0.448
Harrell’s Index of concordance=0.90			

HRs in multivariate model generated for outcomes of increase in fibrosis on follow on CT scan in three models as described in three models—model A—increase in TLF≥7.8 %/litre, (model B) increase in fibrosis≥10% /litre and (model C) relative decline in absolute FVC>10%. Change in lung volume is measured between CT1 and CT2. Leucocyte levels are presented as continuous variables (outcome for dichotomised values are shown in online supplemental table 3).

Values in bold signifies p<0.05.

FVC, forced vital capacity; PVV, pulmonary vessel volume; TLF, total lung fibrosis.

In model C (where outcome was change by 10% of FVC), no leucocyte variable significantly associated with FVC decline. A lower baseline FVC was predictive of FVC decline (HR 0.96, 95% CI 0.92 to 0.99, p=0.049).

All leucocyte derived indexes were significantly associated with progression in fibrosis (TLF/litre by 7.8% and 10%) in multivariate analysis (online supplemental table 3).

50.7% of patients who underwent repeat CT were receiving antifibrotics at first CT. When adjusted for antifibrotic use and comorbidities, significance was preserved for neutrophils, NLR and SIRI (online supplemental tables S4,S5).

### Association between blood leucocyte levels and mortality

**Figure 1:**
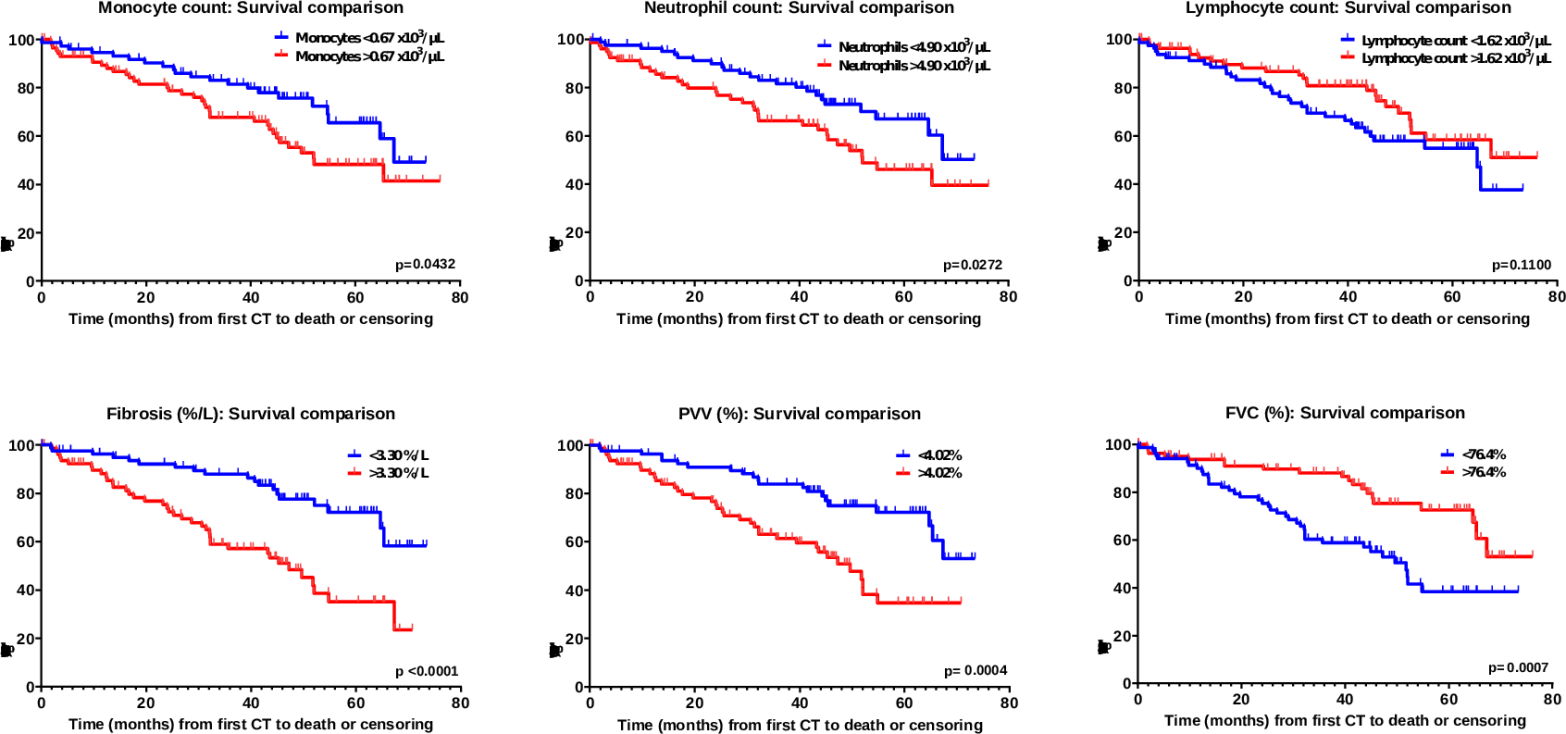
Kaplan-Meier curves for time to mortality for monocyte, neutrophil, lymphocyte levels, TLF, PVV and FVC, all at baseline. Leucocyte levels and CALIPER variables were dichotomised by median value. CALIPER, Computer-Aided Lung Informatics for Pathology Evaluation and Rating; FVC, forced vital capacity; PVV, pulmonary vessel volume; TLF, total lung fibrosis.

During the study period, 60 all-cause deaths (35.1%) were reported in this cohort. Leucocyte levels were dichotomised by median values (figure 1) or normal reference range limits (online supplemental figure S1). Significantly shorter survival times were observed for cases dichotomised by median monocyte count (p=0.033) and neutrophil levels (p=0.0180) (figure 1), and for cases dichotomised by median NLR, MLR and SIRI values (online supplemental figure S1). Shorter survival times were also observed for those with TLF, PVV and FVC greater than the median value (TLF>3.30%/L, p<0.001; PVV>4.02%, p<0.001 and FVC>76.6%, p<0.001) (figure 1).

## Discussion

In this study, we showed that when progression of disease is categorised specifically by increase in amount of fibrosis, quantified by an automated quantitative scoring modality, neutrophil levels rather than monocytes were the key immune correlate with progression in amount of fibrosis in the lungs. MLR, NLR and SIRI were also associated with progression. Although monocyte count was not associated with disease progression in this cohort, importantly, as with other studies, higher monocyte levels in our cohort were associated with mortality.^4 21^ Our findings suggest that neutrophils may be a contributor to active accumulation of fibrosis in IPF, supported by previous findings of high neutrophils in bronchoalveolar lavage (BAL) of IPF patients,^22^ and more recent findings of higher NLR in IPF patients with greater rates of FVC decline.^5^

Neutrophils have long been associated with immunopathogenesis of IPF. Neutrophilia in BAL specimens from IPF patients is associated with earlier mortality.^22^ Neutrophil elastases (NE) are elevated in IPF BAL samples,^23^ and experimental data using murine models suggest that NE activates transforming growth factor-ß pathway and fibroblast proliferation.^24^ An intriguing and newly identified fibrosis-promoting function of neutrophils is generation of neutrophil extracellular traps (NETs).^25^ These proinflammatory collections of chromatin and neutrophils regulate both immune cell function and fibroblast activation.^26^ While a specific association with IPF has yet to be fully described, enhanced detection of intrapulmonary NETs has been reported in bleomycin models and in non-IPF fibrotic ILD studies.^27 28^ Here, we are able to link blood neutrophil level measured at baseline CT specifically with progression in amount of fibrosis over time. Further prospective translational studies are required to establish the regulatory role of neutrophils, NET formation over time and cytokine and chemokine activity at different stages of fibrosis.

In addition to TLF change, PVV measures also correlated with neutrophil count. PVV is an intriguing quantitative variable that has proved a consistent predictor of disease severity, progression and mortality in ILD studies.^12 14 29–31^ Our findings are supportive of the original work performed by Jacob *et al* who demonstrated that PVV% was independently associated with FVC decline,^32^ and Chung *et al* who demonstrated PVV negatively correlated with TLCO%.^33^ In our study, PVV was independently associated with progression of fibrosis. The pathological mechanisms linking PVV with adverse ILD outcomes are not fully understood and several theories have been suggested. Blood perfusion is reduced in areas of pulmonary fibrosis,^34^ but increased in adjacent areas of unaffected lung.^35^ Jacob *et al* postulated that correlation between ILD extent and vessel calibre may represent regional elevation in pulmonary artery pressures in mildly fibrotic lung or destruction of the capillary bed in more advanced disease leading to neovascularisation and diversion of blood to unaffected lung areas.^12^ Another possible explanation relates to the negative intrathoracic pressures required of non-compliant fibrotic lungs to generate adequate inspiratory volumes. This could in turn exert additional ‘tractional’ force on the lung vasculature resulting in dilatation in fibrotic regions in a traction-like phenomenon, akin to traction bronchiectasis.^33^

The trend of lower lymphocytes count with progression of fibrosis mirrors the findings of previous studies.^5 36^ Lymphocytic aggregates are a recognised pathological feature of IPF lesions.^27 37^ The association between low blood lymphocyte count and adverse outcomes in IPF is currently unknown but could be explained in part by lymphocyte dysfunction,^38^ and the sequestering of lymphocytes into sites of inflammation, such as the fibrotic lung. We note that post hoc analysis of the ASCEND and CAPACITY studies Nathan *et al* reported that serial increase in NLR over 12 months was associated with mortality.^39^

In our study, we report association between higher neutrophil count and lower lymphocyte count taken from full blood count analysis with progression of fibrosis scoring on HRCT. To our knowledge, this specific association has not been reported elsewhere. Other published studies and post hoc analyses have only reported leucocyte association with mortality, hospitalisation and FVC decline.

Our findings should be framed within the following limitations. First, the retrospective nature of the study meant that we selected patients who had repeat CT scans, which were clinically indicated. This introduced a selection bias towards patients who had a clinical reason to have a CT scan—often, this is worsening in disease either over time or acutely, and CT scans would be performed at different/non-uniform time intervals. Unfortunately, we did not have access to blood leucocyte data measured at the time of the second CT scan. Analysing blood leucocytes/ratios at second CT could may have provided additional information, especially with regard to how any relative change in blood count/ratio may differ between patients with progression of fibrosis or stability at second CT scans. Although the leucocyte levels were measured at the time of the first (presenting) CT scans, this group of patients could have a different disease trajectory compared with those who did not have a second CT scan. There is a possibility that neutrophil levels are more likely a driver in those who are clinically deteriorating. The baseline correlation data (n=171 patients), however, are not limited by these factors. Further analysis of prospective validation cohorts would be required to investigate whether neutrophil count, measured in peripheral blood, could identify patients at greater risk of progression of fibrosis in IPF, and consequently facilitate prognostication of the disease.

From the point of fibrosis scoring, the sum of GGO, reticulation and honeycombing was used as the TLF score.^14^ Although each scan was reported by a radiologist who ‘called’ the GGO as fine fibrosis and the overall pattern as probable or definite UIP, other causes such as pulmonary oedema, acute exacerbation and infection could have contributed to this CT finding.^40^

Nevertheless, our study shows that greater neutrophil count was significantly associated with quantifiable increase in fibrosis on imaging of the lungs in IPF. Further studies will be required to validate this finding.

## Data Availability

Data are available on reasonable request. N/A.
